# A Functional Connectome of Parkinson's Disease Patients Prior to Deep Brain Stimulation: A Tool for Disease-Specific Connectivity Analyses

**DOI:** 10.3389/fnins.2022.804125

**Published:** 2022-06-24

**Authors:** Aaron Loh, Alexandre Boutet, Jürgen Germann, Bassam Al-Fatly, Gavin J. B. Elias, Clemens Neudorfer, Jillian Krotz, Emily H. Y. Wong, Roohie Parmar, Robert Gramer, Michelle Paff, Andreas Horn, J. Jean Chen, Paula Azevedo, Alfonso Fasano, Renato P. Munhoz, Mojgan Hodaie, Suneil K. Kalia, Walter Kucharczyk, Andres M. Lozano

**Affiliations:** ^1^Krembil Neuroscience Center, University Health Network, Toronto, ON, Canada; ^2^Joint Department of Medical Imaging, University of Toronto, Toronto, ON, Canada; ^3^Movement Disorders and Neuromodulation Unit, Department of Neurology With Experimental Neurology, Charité Universitätsmedizin Berlin, Corporate Member of Freie Universität Berlin, Humboldt-Universität zu Berlin, and Berlin Institute of Health, Berlin, Germany; ^4^Baycrest Health Sciences, Rotman Research Institute, Toronto, ON, Canada; ^5^Department of Medical Biophysics, University of Toronto, Toronto, ON, Canada; ^6^Division of Neurology, Edmond J. Safra Program in Parkinson's Disease, Morton and Gloria Shulman Movement Disorders Clinic, Toronto Western Hospital, University Health Network, University of Toronto, Toronto, ON, Canada; ^7^Krembil Brain Institute, University Health Network, Toronto, ON, Canada; ^8^Center for Advancing Neurotechnological Innovation to Application (CRANIA), Toronto, ON, Canada; ^9^Division of Neurosurgery, Department of Surgery, University of Toronto, Toronto, ON, Canada

**Keywords:** Parkinson's disease, functional connectivity, connectomics, neuromodulation, functional magnetic resonance imaging

## Background and Summary

A wide range of disorders are thought to arise from dysfunction in brain circuitry (Bonelli and Cummings, 2007). These pathological circuits are not directly appreciated on routinely acquired structural MRI sequences. In contrast, functional sequences, such as resting state functional magnetic resonance imaging (rs-fMRI), allow us to probe networks and generate a “connectome” that facilitates a global assessment of brain circuitry and function (Yeo et al., 2011). The dearth of patient-specific, or “native”, functional imaging in the majority of clinical protocols and the limited reliability of individual acquisitions has led investigators to instead use large, publicly available aggregates of rs-fMRI (i.e., normative connectomes) to examine brain connectivity and study relationships between connectivity and clinical outcome (Supplementary Table 1) (Fox, 2018). However, a common limitation is that these datasets are often derived from healthy subjects. Differences between the inherent connectivity of the healthy and diseased brain mean that these normative connectomes may not be optimal to study brain circuits in diseased populations (Sala et al., 2017). Even while initial studies made cursory efforts at using disease-matched connectomes (Horn et al., 2017), it may be crucial to match disease-severity and patient age as closely as possible to the patient collective of study. For instance, if the connectomes would be used in the context of Parkinson's Disease (PD) patients undergoing deep brain stimulation (DBS), it would be most optimal to acquire the connectome within exactly such a sample of patients. This was the motivation for constructing the present dataset.

Thus far, normative connectomes have mostly been used to map specific neurological symptoms or phenomena to brain circuits (Fox, 2018) and to define engaged networks in patients undergoing neuromodulation (Fox et al., 2014). Using brain lesions, this approach has been applied in numerous recent studies to map phenomena such as free will (Darby et al., 2018), aggression (Yan et al., 2020), and depression (Padmanabhan et al., 2019). The DBS field has leveraged normative connectomics to identify networks mediating clinical benefits. DBS is a neurosurgical treatment in which electrodes are implanted into precise brain structures to deliver electrical stimulation and provide clinical benefits (Lozano and Lipsman, 2013), with more than 200,000 individuals implanted worldwide (Hariz, 2017). DBS is best established as a therapeutic tool for PD and other movement disorders such as essential tremor and dystonia, while also being investigated as a treatment for psychiatric and cognitive disorders (Harmsen et al., 2020). To date, analyses with normative connectomes have characterized networks critical for symptom alleviation in DBS treatment of Parkinson's disease (PD) (Horn et al., 2017) and obsessive compulsive disorder (Baldermann et al., 2020).

Such studies have used normative connectomes because of (1) a lack of native (i.e. patient-specific) functional imaging, (2) limited test-retest reliability in intra-individual acquisitions (meaning that single acquisitions are inherently less reliable than aggregates) (Fox et al., 2013; Holiga et al., 2018), and (Fox, 2018) the state-of-the-art MRI hardware and acquisition parameters used in constructing certain normative connectivity datasets (Yeo et al., 2011; Horn et al., 2017). Nevertheless, in this type of analysis, the specific circuit organization of each individual is necessarily obscured. Attaining optimal individual data, however, requires long scanning time and repeated acquisitions, which may be impractical, if not impossible, with frail diseased populations. Given the relative attributes of existing normative connectomes and native functional imaging, disease-specific aggregates of functional imaging (i.e., disease-specific normative connectomes) may offer a valuable compromise (Wang et al., 2020).

As PD is the most common DBS indication, we aimed to acquire native rs-fMRI in a large cohort of PD patients using acquisition parameters practical in this patient population. The rs-fMRI acquisitions were incorporated into a PD-specific functional connectome which may now be used as a tool to investigate the mechanisms of action of neuromodulatory treatments, as well as the network-level underpinnings of PD itself. The connectome is completely pre-processed and in a format that allows easy access using published open-source software (Horn et al., 2019). Herein, we demonstrate the merits of using a disease-specific connectome, showing that the PD-specific connectome is reliable and that it is able to offer unique insights–not otherwise achievable with a healthy normative connectome–when performing certain PD-specific functional connectivity analyses.

## Methods

### Creating the PD-Specific Connectome

#### Participants

Eighty patients underwent preoperative MRI for DBS target planning between June 2018 and August 2020 at Toronto Western Hospital, Toronto, Canada. Research ethics board (REB) approval (REB #14-8255) and informed consent was acquired for the addition of rs-fMRI acquisitions during the preoperative MRI. The work described was performed in accordance with the Declaration of Helsinki. Included patients had a clinical diagnosis of idiopathic PD and were awaiting DBS implantation of either the subthalamic nucleus (STN) or internal globus pallidus (GPi). Patients with confounding neurological comorbidity (e.g., space occupying lesion, stroke, or multiple sclerosis) were excluded from the study. Demographic and clinical information for these patients can be seen in Table 1.

**Table 1:** Demographic information of pre-DBS PD patients included in the Tor-PD connectome.

	**Age (years)**	**Sex**	**Disease duration (years)**	**Pre-op MDS-UPDRS**	**Pre-op Levodopa equivalent (mg/day)**	**Active contact coordinates (mm)**
Tor-PD cohort (*n* = 75)	62 ± 9	44 M 37 F	11 ± 4	71 ± 30	1,131 ± 609	N/A

*Values represent the mean and standard deviation*.

*DBS, deep brain stimulation; F, female; M, male; MDS-UPDRS, Movement Disorders Society - Unified Parkinson's Disease Rating Scale; mg, milligram; PD, Parkinson's disease*.

#### Imaging Protocol

Included patients underwent MRI prior to their DBS surgery. For each patient, a T1-weighted 3D spoiled gradient echo recall (SPGR) sequence and a gradient echo (GRE) echo planar-imaging (EPI) rs-fMRI sequence (duration = 6.5 min) were acquired. MRI acquisition parameters for each sequence can be obtained from Supplementary Table 1. Patients underwent scanning in the medication ON state (i.e., without withholding their regular dose of levodopa or dopamine agonist) to minimize movement in the MRI. For rs-fMRI acquisitions, patients were asked to lay still, keep their eyes closed, and think of nothing in particular. Two patients consented to undergo a second rs-fMRI acquisition to add to the size of the dataset, for a total of 82 rs-fMRI acquisitions. Five scans were removed due to suboptimal acquisitions. Therefore, 77 raw rs-fMRI scans (58 at 3T and 19 at 1.5T) were included for pre-processing (Supplementary Figure 1).

#### fMRI Preprocessing

Preprocessing steps paralleled those employed in constructing a commonly used healthy normative connectome using Brain Genomics Superstruct Project (GSP) data (https://dataverse.harvard.edu/dataverse/GSP) (Yeo et al., 2011), with additional steps included to mitigate the effects of potentially increased motion in our PD cohort. These steps were performed using tools from the FMRIB Software Library (FSL 5.0) (https://fsl.fmrib.ox.ac.uk) (Jenkinson et al., 2012) and Analysis of Functional NeuroImages (AFNI-vAFNI_2011_12) (https://afni.nimh.nih.gov) (Cox, 1996) library. Individual preprocessing steps are detailed in the Supplementary Material, and a visual schematic of the pipeline can be seen in Supplementary Figure 2.

#### Generating a Useable and Publicly Accessible Tool

To facilitate group-level analyses, the preprocessed rs-fMRI data was transformed into ICBM 2009b nonlinear asymmetric (MNI 152 ‘standard') space using Lead-DBS (http://lead-dbs.org/) (Horn et al., 2019). This was performed using Advanced Normalization Tools (https://github.com/ANTsX/ANTs). The structural data (i.e. T1-weighted scan) was non-linearly transformed to standard space, after which the ensuing transform was applied to the rs-fMRI data. This was performed with the “Effective: low variance” preset, which has been shown to be the most accurate spatial normalization technique amongst other commonly used normalization algorithms (Ewert et al., 2019). Following normalization of each fMRI acquisition, a 300,000 x 175 matrix–containing the BOLD signal of every voxel (*n* = 300,000) for each volume in the time series (*n* = 175)–was computed using the program Lead Connectome (https://www.lead-dbs.org) (Horn et al., 2019).

**Figure 1:**
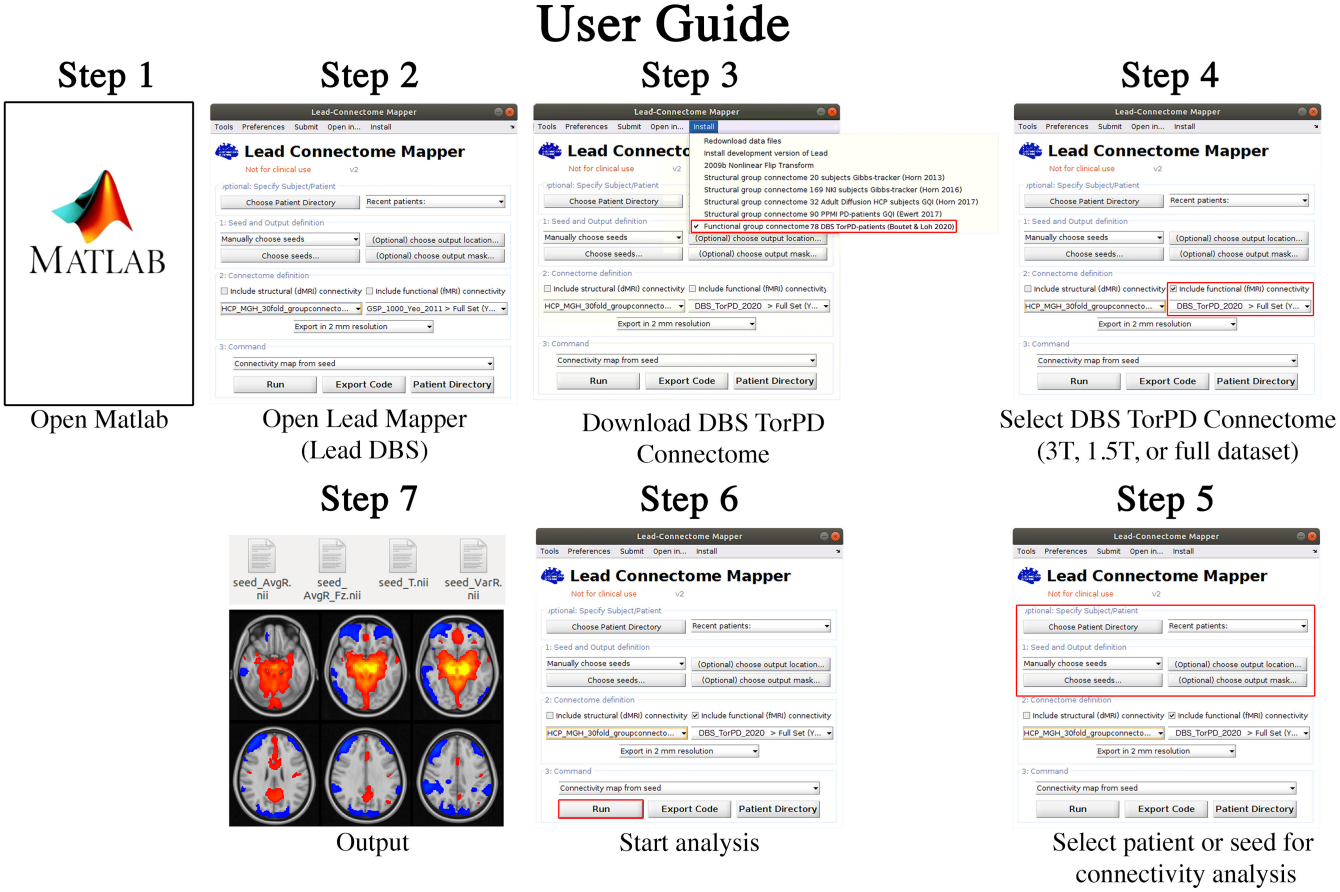
Tor-PD User Guide. A visual guide detailing how to use the Tor-PD connectome to perform normative functional connectivity analyses. Step 1: Open Matlab. Step 2: In Matlab, open Lead Mapper. Step 3: Install the Tor-PD connectome. Step 4: Check the box ‘include functional (fMRI) connectivity' and select ‘DBS_TorPD_2020' from the dropdown menu. Step 5: Select the patient or seed for connectivity analysis. Step 6: Run the analysis. Step 7: The analysis will output four NIfTI files showing connectivity of the seed to the rest of the brain. DBS, deep brain stimulation; fMRI, functional magnetic resonance imaging; NIfTI, neuroimaging informatics technology initiative; T, tesla.

**Figure 2:**
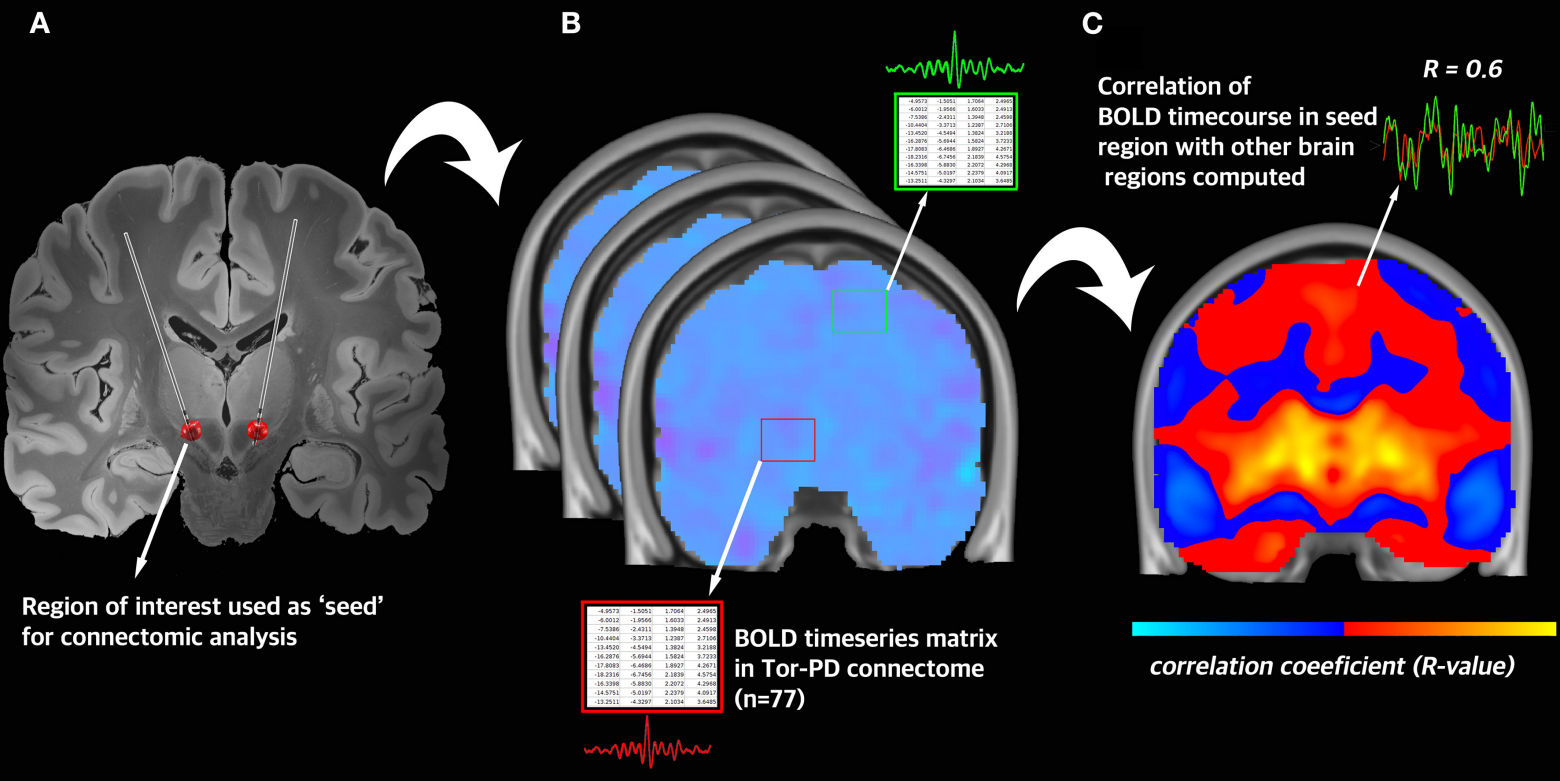
Visual schematic of connectomic analysis with the Tor-PD connectome. **(A)** A region-of-interest is defined and used as a seed in connectomic analysis. In this example, the seed is the volume of tissue activated by DBS. **(B)** Across all n = 77 rs-fMRI acquisitions in the Tor-PD connectome, the BOLD timeseries is extracted from the seed region and a separate region in the brain. **(C)** The average correlation coefficient is computed between the BOLD timeseries in each region. This correlation coefficient represents the strength of functional connectivity between these regions. BOLD, blood-oxygen-level-dependent signal; DBS, deep brain stimulation; rs-fMRI, resting state functional magnetic resonance imaging.

The resultant 77 BOLD signal time-series matrices–corresponding to the 77 rs-fMRI acquisitions–were collated into a single dataset, which we have named the Toronto-Parkinson's Disease (Tor-PD) connectome. In addition, we also created a mask corresponding to the voxels in standard space where BOLD signal could be calculated in at least 80% of the 77 matrices **(****Supplementary Figure 4****)**. This mask can be applied to maps computed using the Tor-PD connectome if users wish to stringently remove areas of relatively limited data (i.e. areas where voxels had signal dropout >20% of the time, such as the orbitofrontal cortex) from their analysis (Supplementary Figure 4). The Tor-PD connectome and mask were subsequently uploaded to Zenodo (https://doi.org/10.5281/zenodo.4310183) and Lead-DBS (https://www.lead-dbs.org/) for public access and use. A visual user-guide for investigators can be seen in Figure 1, a visual schematic of the connectome can be seen in Figure 2, and usage notes can be found in the Supplementary Material (Usage Notes).

## Validation

### Functional Connectivity Analyses

To investigate its overall reliability and utility within the connectomic field, we performed three exemplar connectivity analyses using the Tor-PD dataset. First, we assessed whether we were able to identify the default mode network (DMN)–a canonical brain network that shows increased BOLD response during wakeful rest–in the Tor-PD connectome. Second, to assess for gross differences in PD DBS target connectivity, we compared the whole-brain functional connectivity of the subthalamic nucleus (STN), internal globus pallidus (GPi), and ventral intermediate nucleus (VIM) of the thalamus when computed using the Tor-PD connectome and a commonly used connectome composed of 1,000 rs-fMRI acquisitions acquired within the Brain Genomics Superstruct Project (https://dataverse.harvard.edu/dataverse/GSP) (healthy adults; 42% males; mean age = 23 years; range = 18–35 years) (Yeo et al., 2011). Henceforth, we will refer to the latter as the Healthy connectome. Finally, we compared how well the Tor-PD and Healthy connectome performed when functional connectivity between DBS sites and motor regions of interest was used to explain variance in individual DBS outcomes in PD patients. All statistical analyses were performed using R (R3.4.4, https://www.r-project.org) and RMINC (https://github.com/Mouse-Imaging-Centre/RMINC).

### Default Mode Network Connectivity

The default mode network (DMN) is a canonical large-scale brain network consisting of distributed nodes that show increased and correlated BOLD response during wakeful rest (Raichle et al., 2001). Consequently, it should be readily identifiable in the Tor-PD connectome. To assess this, we used a node of the DMN [specifically the dorsal medial prefrontal cortex (Shirer et al., 2012)] as a seed in the Tor-PD connectome to derive a whole brain connectivity (or raw *r-*) map. All raw *r*-maps described herein were computed using the Lead Connectome Mapper software; https://www.lead-dbs.org) (Figure 1). This map shows average functional connectivity estimates (correlation coefficients) between the seed and every voxel in the brain based on low-frequency BOLD signal fluctuations sampled across the 77 fMRI acquisitions. To discern meaningful connections, we transformed the r to *t*-values, then thresholded and binarized to only show voxels with a t value of > 2.8 (*p* = 0.001, uncorrected). Finally, we compared the similarity of our thresholded and binarised t-maps, and a publicly available mask of the DMN (Shirer et al., 2012). To do this, we computed their Dice similarity coefficient (DSC) using the formula:


$$\begin{array}{l} DSC\;=\;\frac{2|X\cap \;Y|}{\left|X|+|Y\right|} \end{array}$$

In which |X| and |Y| are the cardinalities of the two sets (i.e., the number of elements in each set).

**Figure 3:**
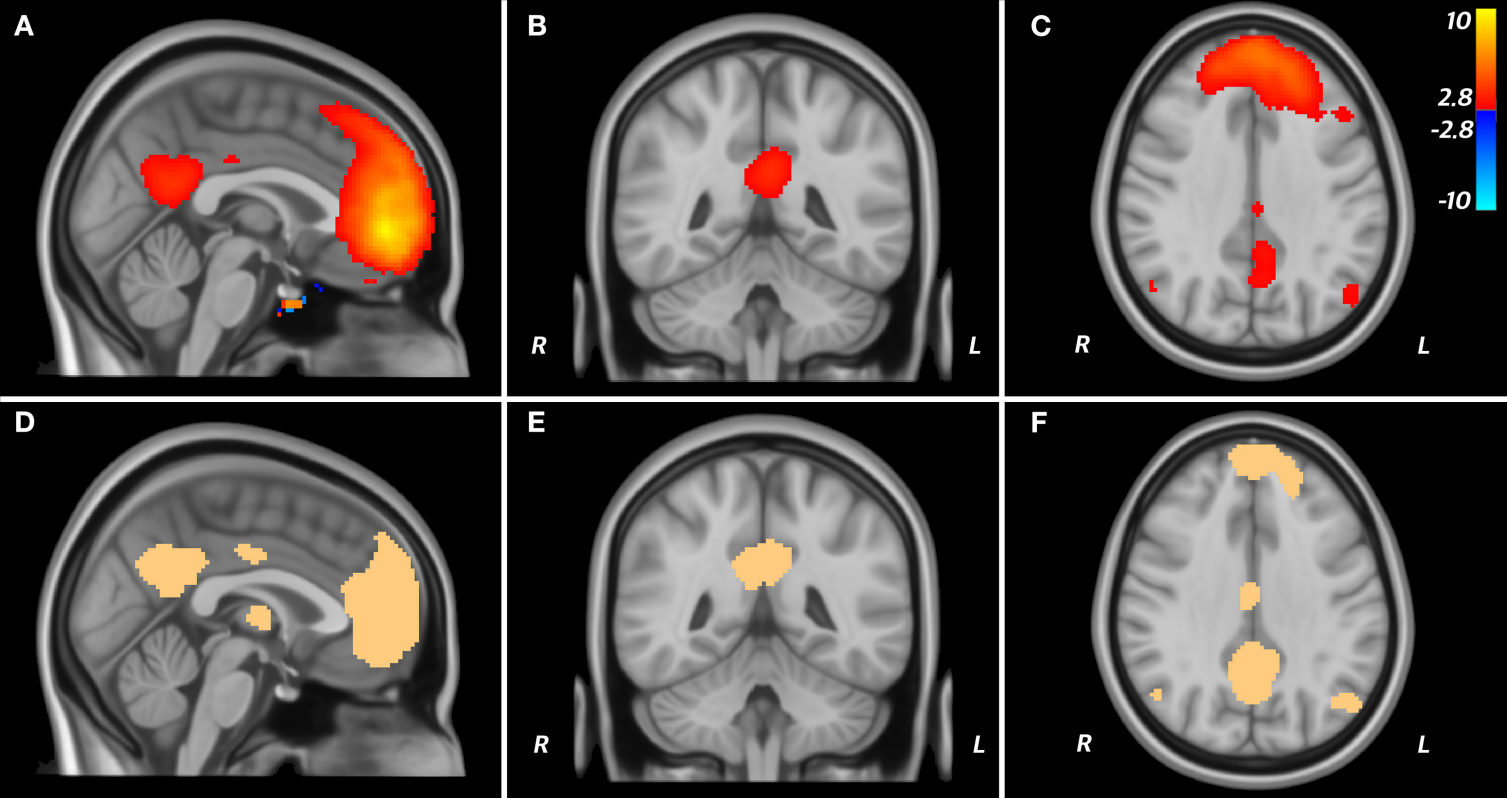
DMN in the Tor-PD connectome. A T map displaying areas functionally connected (T > 2.8; P < 0.001) to the dmPFC when seeded in the Tor-PD connectome **(A–C)** is shown alongside a mask of the DMN **(D–F)**. These areas are shown overlaid on sagittal **(A,D)**, coronal **(B,E)**, and axial **(C,F)** slices of a T1-weighted standard brain template (MNI ICBM 2009b NLIN asymmetric). On the T map, “hot” colors represent areas where the BOLD signal is positively correlated with the signal in the seed, while “cold” colors represent ante-correlation. BOLD, blood-oxygen-level-dependent; DMN, default mode network; dmPFC, dorsal medial prefrontal cortex; ICBM, International COnsortium for Brain Mapping; MNI, Montreal Neurological Institute; NLIN, non-linear.

The thresholded (*t* > 2.8; *p* = 0.001, uncorrected) and binarised t-map derived from seeding the dorsal medial prefrontal cortex in the Tor-PD connectome included the posterior cingulate cortex and the angular gyrus, comprising the remaining nodes of the DMN (Figures 3A–C). Further, the Dice similarity coefficient (DSC) of this t-map and a publicly available mask of the DMN (Figures 3D–F) reflected good spatial agreement (DSC = 0.58), while 68% of the t-map was contained within the DMN mask.

### Connectivity of DBS Targets in the Tor-PD Connectome and Healthy Connectome

We compared the whole-brain functional connectivity of PD-DBS targets, namely the (i) STN (Horn et al., 2017), (ii) GPi (Horn et al., 2017), and (iii) VIM (Horn et al., 2017)–within the Tor-PD and Healthy connectomes. DBS targets were used as seeds in each connectome, resulting in two whole brain-connectivity raw *r*-maps for each DBS target (corresponding to either the Tor-PD or Healthy connectome). To compare the raw *r*-maps computed by either connectome, we performed mass-univariate testing using false discovery rate to correct for multiple comparisons. We also examined the effect of different MRI hardware, performing the same comparison but with a subset (*n* = 58) of the Tor-PD connectome using 3T rs-fMRI scans only (i.e., excluding acquisitions performed with 1.5T MRI).

When seeding common PD DBS targets in the Tor-PD connectome and Healthy connectome, there was a negligible number of significantly different (p_FDRcorrected_ < 0.05, voxel-wise, thus of every 100 significant voxels 5 might be false positive) voxels across the whole brain (300,000 voxels) between raw r-maps computed using either connectome (seven significantly different voxels when the STN was seeded, five when the VIM was seeded, and two when the GPi was seeded). Moreover, when we examined the effect of different MRI hardware, the number of significantly different voxels between raw *r-*maps from either connectome was the same when computed with the full Tor-PD connectome or a subset of the Tor-PD connectome using 3T rs-fMRI acquisitions (58 scans) only.

### The Relationship Between Functional Connectivity and Clinical Outcomes in DBS Patients

Finally, we compared the degree to which functional connectivity between individual DBS stimulation areas and motor regions could explain variance in clinical outcome when computed using the Tor-PD connectome or Healthy connectome. Using the Healthy connectome, previous studies have shown that the functional connectivity between the local area around the electrode modulated by DBS (estimated by a model termed *volume of tissue activated*; VTA) and the rest of the brain are predictive—across DBS cohorts and centers—of DBS outcome in PD patients (Horn et al., 2017). To demonstrate the potential utility of our connectome in such a research setting, we performed this analysis with VTAs and clinical outcomes in a random selection of 50 STN-DBS patients from our center using the Tor-PD connectome and the Healthy connectome. These patients were independent from those used to make the Tor-PD connectome, and are described in detail by Elias and colleagues in a previous study (Elias et al., 2020). VTA patient demographics and associated clinical outcomes from DBS (percentage Unified Parkinson's Disease Rating Scale (UPDRS) III change) can be seen in Supplementary Table 2. As reported elsewhere, stimulation parameters at the time of each patient's best motor response to DBS were used to construct individual VTAs (Elias et al., 2020). VTA modeling was performed using Lead DBS according to previously published methods (Horn et al., 2017; Elias et al., 2020). Bilateral VTAs (from each patient's left and right electrode) were used as seeds in each connectome, resulting in a corresponding raw *r*-map for each patient's seeded VTA. We then extracted the average correlation between each pair of bilateral VTAs and regions-of-interest (ROIs) that have previously been implicated in motor improvement in PD (Akram et al., 2017; Horn et al., 2017)—the bilateral primary motor cortex (M1) (Mayka et al., 2006), premotor cortex (PM) (Mayka et al., 2006), supplementary motor area (SMA) (Mayka et al., 2006), and cerebellum (Diedrichsen, 2006). Subsequently, a linear model was calculated to investigate the relationship between VTA-ROI functional connectivity and individual patient outcomes. We then compared each patient's predicted improvement based on these linear models to actual outcome.

**Figure 4:**
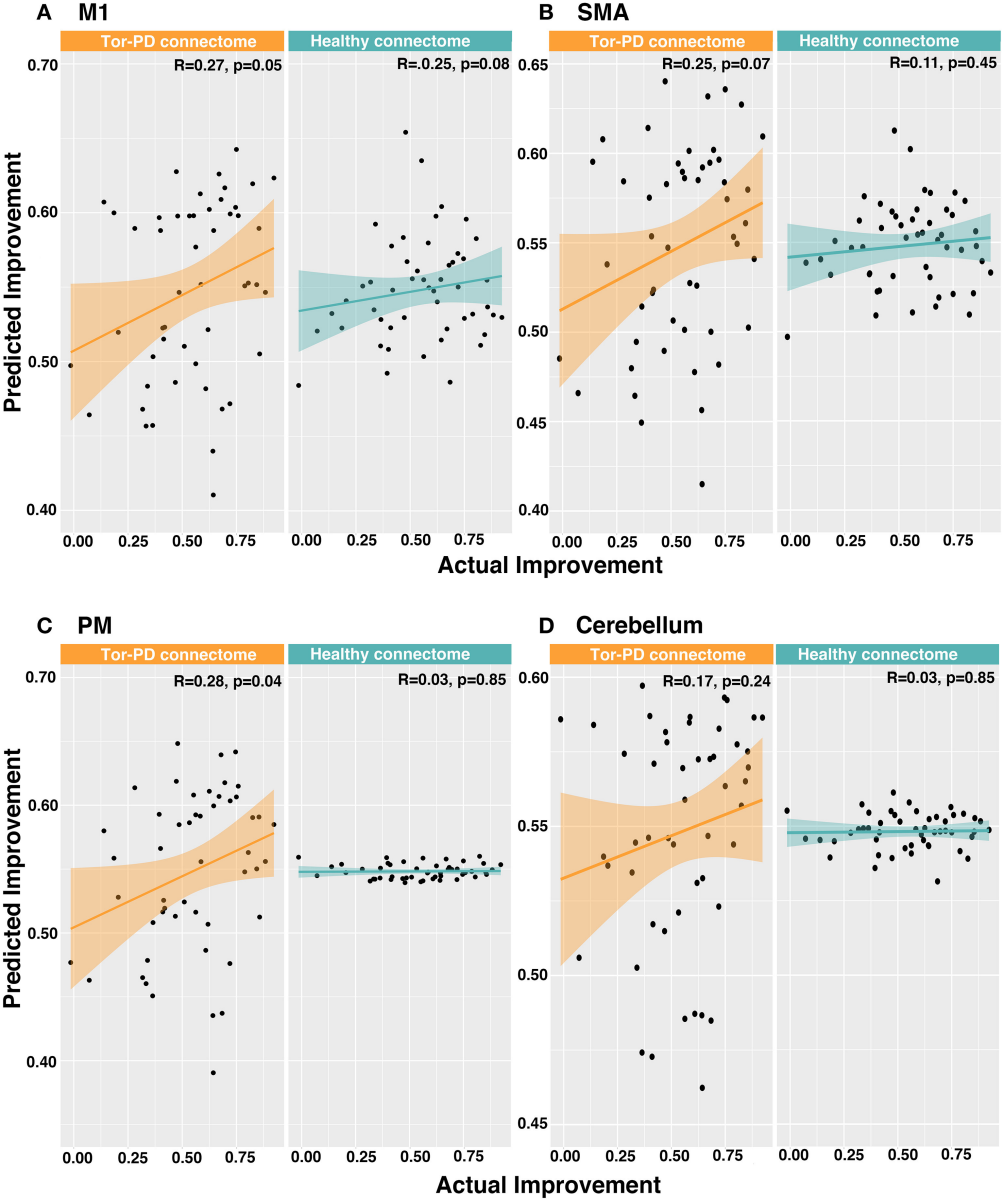
Predicting DBS outcome in PD patients based on functional connectivity of VTAs to motor ROIs. Actual vs. predicted improvement based on functional connectivity of VTAs to M1 **(A)**, SMA **(B)**, PM **(C)**, and cerebellum **(D)**. DBS, deep brain stimulation; M1, primary motor cortex; SMA, supplementary motor area; PD, Parkinson's disease; PM, premotor cortex; VTAs, volumes of tissue activated.

When investigating functional connectivity between VTAs and motor ROIs, we found that connectivity of VTAs to M1 (R = 0.27, *p* = 0.05) and PM (R = 0.28, *p* = 0.04) could significantly explain outcome when using the Tor-PD connectome, but not the Healthy connectome (M1: R = 0.16, *p* = 0.27; PM: R = 0.03, *p* = 0.85). Functional connectivity between VTAs and SMA or cerebellum could not significantly explain outcome when using either connectome (Figures 4A–D). Finally, a combined linear model incorporating functional connectivity of VTAs to all four motor ROIs could significantly explain outcome using the Tor-PD connectome (R = 0.3, *p* = 0.04), but not the Healthy connectome (R = 0.25, *p* = 0.08).

## Summary and Limitations

Using rs-fMRI from 75 PD patients, we created a freely accessible and user-friendly tool that allows investigators to readily perform PD-specific functional connectomic analyses (Figure 1). In our first connectivity analysis, we showed that the DMN is identifiable in the Tor-PD connectome, pointing to the overall reliability of the data. Then, we demonstrated that normative connectomic analyses using the Tor-PD connectome can lead to findings that are comparable to those obtained using the Healthy connectome, which is the most commonly used connectome in modern network mapping (Horn and Fox, 2020). The similarity of these findings—one achieved with a disease-specific dataset and one achieved with a very large dataset of healthy individuals—reinforce the reliability of our data, while also substantiating the findings of previous studies using the Healthy connectome. Finally, we showed that despite the considerable disparities in size of the Tor-PD (*n* = 75) and Healthy connectomes (*n* = 1,000), when performing analyses with PD DBS patient data, functional connectivity of VTAs to certain motor ROIs computed with the Tor-PD connectome could significantly explain variance in clinical outcome, whereas connectivity computed with the Healthy connectome could not. The adoption of the Tor-PD in future studies, which we have shown can deliver comparable and potentially unique insights relative to healthy normative connectomes, may ultimately engender progress in the understanding and treatment of PD. For example, analyses with healthy connectomes have shown that targets for invasive and non-invasive neuromodulatory therapies for depression map to common brain networks (Padmanabhan et al., 2019). While there is currently a paucity of effective non-invasive neuromodulatory therapies for PD, the Tor-PD connectome could be used in a similar vein to identify PD-specific brain networks that may yield novel (i.e., beyond STN and GPi), effective, and accessible targets for non-invasive treatments. Crucially, the format of the Tor-PD connectome means that further rs-fMRI acquisitions from PD DBS patients can be readily added in the future to grow and further enhance the utility of the tool.

It is important to acknowledge certain limitations of the Tor-PD connectome. Firstly, the connectome is composed entirely of patients who were due to undergo deep brain stimulation. It is important to note that these patients are therefore not representative of the whole PD population. Secondly, while it is the largest disease-specific connectome of its kind, it is composed of considerably fewer rs-fMRI acquisitions than the Healthy connectome. Moreover, comparison with other existing connectomes is inherently limited by differences in MRI hardware, acquisition parameters, and preprocessing. Nonetheless, previous groups have investigated the influence of connectome size, showing that *r*-maps obtained with the healthy connectome (*n* = 1,000) and an earlier iteration (*n* = 98) were nearly identical (Darby et al., 2018). Further, the same group also showed that comparable results can be obtained with different preprocessing strategies (Boes et al., 2015). We also explored the impact of different MRI hardware, finding that *r-*maps computed using a subset of the connectome acquired with 3T MRI only (*n* = 58) had no areas of significant difference compared to those acquired with the full Tor-PD connectome (acquired with both 3T and 1.5T). Future versions of the Tor-PD connectome, or the advent of new disease-specific connectomes, should focus on increasing the number of rs-fMRI acquisitions and ensuring the homogeneity of the MRI hardware, sequence parameters, and preprocessing steps used.

## Conclusion

Overall, our findings demonstrate that the readily accessible Tor-PD connectome is a robust alternative to existing datasets, while underlining the value of using disease-specific connectomes when performing connectivity analyses in PD populations.

## Data Availability Statement

The datasets presented in this study can be found in online repositories. The names of the repository/repositories and accession number(s) can be found below: https://doi.org/10.5281/zenodo.4310183.

## Ethics Statement

The studies involving human participants were reviewed and approved by Research Ethics Board - University Health Network. The patients/participants provided their written informed consent to participate in this study.

## Author Contributions

ALoh, AB, JG, and GE conceived the study. ALoz, MH, SK, AF, PA, MP, and RM informed and facilitated participation selection, recruitment, and consent. ALoh, AB, RG, GE, and CN performed data acquisition. JK, JC, BA-F, and AH guided image preprocessing. JG, BA-F, and ALoh performed the data analysis. RP and EW contributed to figures. ALoh and AB wrote the manuscript. ALoz supervised the project. All authors edited and approved the final version of the manuscript.

## Funding

This work was supported by the German Research Foundation (Deutsche Forschungsgemeinschaft, DFG NE 2276/1-1) (CN), RR Tasker Chair in Functional Neurosurgery (ALoz), Doctoral Research Grant from the German Academic Exchange Service–DAAD (BA-F), and German Research Foundation (Deutsche Forschungsgemeinschaft, 410169619—Emmy Noether Stipend and 424778381—TRR 295) (AH).

## Conflict of Interest

AF serves as a consultant for Medtronic, Abbott, Boston Scientific, Brainlab, Ceregate, and Medtronic and received research grants, personal fees and non-financial support from Boston Scientific, Brainlab and Medtronic and personal fees from Abbott and Ceregate, all outside the submitted work. SK reports honorarium and consulting fees from Medtronic. ALoz serves as a consultant for Medtronic, Abbott, Boston Scientific, and Functional Neuromodulation. The remaining authors declare that the research was conducted in the absence of any commercial or financial relationships that could be construed as a potential conflict of interest.

## Publisher's Note

All claims expressed in this article are solely those of the authors and do not necessarily represent those of their affiliated organizations, or those of the publisher, the editors and the reviewers. Any product that may be evaluated in this article, or claim that may be made by its manufacturer, is not guaranteed or endorsed by the publisher.

## Abbreviations

DBS: deep brain stimulation
PD: Parkinson's disease
VTA: volume of tissue activated.
