# Supplementary material for: A Functional Connectome of Parkinson's Disease Patients Prior to Deep Brain Stimulation: A Tool for Disease-Specific Connectivity Analyses
Source: Front Neurosci. 2022 Jun 24;16:804125. doi: 10.3389/fnins.2022.804125 (PMC9263841; doi:10.3389/fnins.2022.804125)
Supplement: Supplementary file 1 [file Data_Sheet_1.DOCX]

**Supplementary Materials**

**Supplementary methods:**

Participants:

80 patients underwent preoperative MRI for DBS target planning between June 2018 and August 2020 at Toronto Western Hospital, Toronto, Canada. Research ethics board (REB) approval (REB #14-8255) and informed consent was acquired for the addition of rs-fMRI acquisitions during the preoperative MRI. The work described was performed in accordance with the Declaration of Helsinki. Included patients had a clinical diagnosis of idiopathic PD and were awaiting DBS implantation of either the subthalamic nucleus (STN) or internal globus pallidus (GPi). Patients with confounding neurological comorbidity (e.g. space occupying lesion, stroke, or multiple sclerosis) were excluded from the study. Demographic and clinical information for these patients can be seen in Table 1.

Imaging protocol:

Included patients underwent MRI prior to their DBS surgery. For each patient, a T1-weighted 3D spoiled gradient echo recall (SPGR) sequence and a gradient echo (GRE) echo planar-imaging (EPI) rs-fMRI sequence (duration = 6.5 min) were acquired. MRI acquisition parameters for each sequence can be obtained from Supplementary Table 1. Patients underwent scanning in the medication ON state (i.e. without withholding their regular dose of levodopa or dopamine agonist) to minimize movement in the MRI. For rs-fMRI acquisitions, patients were asked to lay still, keep their eyes closed, and think of nothing in particular. Two patients consented to undergo a second rs-fMRI acquisition to add to the size of the dataset, for a total of 82 rs-fMRI acquisitions. All 82 raw rs-fMRI scans were visually inspected. Five scans were removed due to suboptimal acquisitions. (one scan with large left frontal artefact, one scan which did not acquire the top of the brain, and three scans with corrupted DICOMs). Therefore, 77 raw rs-fMRI scans (58 at 3T and 19 at 1.5T) were included for pre-processing.

fMRI preprocessing:

Preprocessing steps paralleled those employed in constructing a commonly used healthy normative connectome using Brain Genomics Superstruct Project (GSP) data (<https://dataverse.harvard.edu/dataverse/GSP>)[(1)](https://paperpile.com/c/JBEekg/mnsqN), with additional steps included to mitigate the effects of potentially increased motion in our PD cohort. These steps were performed using tools from the FMRIB Software Library (FSL 5.0) (https://fsl.fmrib.ox.ac.uk)[(2,3)](https://paperpile.com/c/JBEekg/WWlSf+T8nnO) and Analysis of Functional NeuroImages (AFNI-vAFNI_2011_12) (https://afni.nimh.nih.gov)[(4)](https://paperpile.com/c/JBEekg/Y2icG) library. A visual schematic of the pipeline can be seen in **Supplementary Fig. 2**.

In order to account for magnetic disequilibrium effects on signal, the first five volumes of all rs-fMRI scans were discarded. Subsequently, each scan was left with 175 volumes. Slice time correction (FSL; https://fsl.fmrib.ox.ac.uk) was applied to the data in an interleaved fashion. Realignment and initial motion correction of the rs-fMRI time series was then performed using mcflirt (FSL; https://fsl.fmrib.ox.ac.uk)[(5)](https://paperpile.com/c/JBEekg/wdPVm). In addition, given the nature of our patient cohort, we censored timepoints in the dataset that were corrupted by excessive motion. These time points were identified, and their effects on the analysis removed, using FSL_motion_outliers (FSL; https://fsl.fmrib.ox.ac.uk) [(6)](https://paperpile.com/c/JBEekg/krVlo). Time points corrupted by excessive motion were defined as those with a mean-squared intensity difference of greater than the threshold (75th centile + 1.5 times the interquartile range) with respect to the reference volume. No more than 18 out of 180 volumes were censored in a given acquisition (mean number censored = 9 ± 4). The average movement for all patients can be seen in **Supplemental Fig. 3.**

The data was then masked to only include voxels within the brain using 3dAutomask (AFNI; <https://afni.nimh.nih.gov>). Spatial smoothing was performed using a Gaussian kernel of 6mm full width at half maximum (AFNI) (https://afni.nimh.nih.gov), after which a high pass filter of 0.01Hz and a low pass filter of 0.08Hz was applied to the data in order to mitigate the effects of scanner drift and high frequency noise fluctuations, respectively (AFNI) (<https://afni.nimh.nih.gov>).

Finally, we regressed out the average BOLD time series over cerebrospinal fluid (CSF) and white matter (WM) [(7)](https://paperpile.com/c/JBEekg/tSo3W). To do this, the corresponding T1-weighted structural image for each patient was segmented using the FreeSurfer volumetric labelling analysis pipeline [(8)](https://paperpile.com/c/JBEekg/NnEnb); [(9)](https://paperpile.com/c/JBEekg/FvjpR). The resultant masks were linearly aligned to the rs-fMRI images with FSL FLIRT [(5)](https://paperpile.com/c/JBEekg/wdPVm) using 6 degrees of freedom, from which masks of WM, CSF and gray matter (GM) were obtained. The average signal over the CSF and WM masks was then calculated and regressed from the rs-fMRI time series via linear regression.

**The relationship between functional connectivity and clinical outcomes in DBS patients using the 3T subset of the Tor-PD connectome:**

When investigating functional connectivity between VTAs and motor ROIs using the 3T subset of the Tor-PD connectome, we found that connectivity of VTAs to PM (R=0.29, p=0.04) was significantly related to outcome, while connectivity to M1 exhibited a trend towards significance (R=0.26, p=0.07). This was not seen with the Healthy connectome (M1: R=0.16, p=0.27; PM: R=0.03, p=0.85). Functional connectivity between VTAs and SMA or cerebellum could not significantly explain outcome when using either connectome. Finally, a combined linear model incorporating functional connectivity of VTAs to all four motor ROIs could significantly explain outcome using the 3T subset of the Tor-PD connectome (R=0.35, p=0.01), but not the Healthy connectome (R=0.25, p=0.08).

**Usage notes:**

Users may choose to download the Tor-PD connectome directly from Zenodo (https://doi.org/10.5281/zenodo.4310183) for their own analysis, or use it as part of a validated semi-automated pipeline in Lead DBS (https://www.lead-dbs.org/).[(10–16)](https://paperpile.com/c/JBEekg/s1LM6+LgtKd+5fOne+IAV24+XQdtC+LOs52+TzsxI) To facilitate general usability, we have enclosed usage notes for the Tor-PD connectome. Investigators may also refer to our visual user guide (**Figure 1**).

To perform functional connectivity analyses using the Tor-PD connectome without custom scripts, users are advised to download Lead-DBS (<https://www.lead-dbs.org/>),[(16)](https://paperpile.com/c/JBEekg/TzsxI) an open-source MATLAB (MATLAB, The MathWorks, Inc. Natick, MA, USA) based toolbox. After opening the Lead Mapper tool included within Lead DBS, users will be able to download the Tor-PD connectome from the ‘install’ dropdown menu. Following installation, users will be able to select seeds to perform seed-to-whole-brain connectivity analyses. Seeds – that must be in NIfTI-1 format – should be binary or weighted regions in standard space (MNI152 space) and may constitute any region of interest in the brain that the users wish to investigate (e.g. VTAs following DBS or lesions following focused ultrasound ablation). The software also supports using weighted activation maps or other regions of interest as seed input. Users are then able to mark the checkbox ‘Include functional (fMRI) connectivity’ and select the Tor-PD connectome. Users start the analysis by pressing ‘Run’, after which Lead Mapper will iteratively compute the correlation of the BOLD values in the seed region and the rest of the brain in all 77 rs-fMRI matrices. Lead Mapper will output multiple maps in NIfTI-1 format corresponding to the voxel-wise average R values (correlations) of the seed and every voxel in the standard brain (MNI152 space), a Fisher-z-transformed version of this map, a T-map across 77 acquisitions, and a map denoting standard deviations.

**Search methods for Supplementary Table 1. *Deep brain stimulation studies investigating relationships between connectivity and movement disorder patient outcomes using normative connectivity analyses.***

A search was conducted on August, 2020 using the PubMed database. The search strategy employed search terms: “Deep Brain Stimulation”, “connectome”, “connectivity”, and “normative”. Only studies using normative connectivity analysis and relating to movement disorders (structural or functional) were included.

**Supplementary Table 1. Deep brain stimulation studies investigating relationships between connectivity and movement disorder patient outcomes using normative connectivity analyses.**

| **Study** | **DBS indication** | **Outcome variable** | **Connectome type*** | **Strength of correlation between DBS site connectivity and outcome** |
| --- | --- | --- | --- | --- |
| Connectivity predicts deep brain stimulation outcome in Parkinson’s disease.[(10)](https://paperpile.com/c/JBEekg/s1LM6) | PD | Motor improvement | Structural (healthy subjects: n=32);  Functional (healthy subjects: n=1000) | healthy structural connectome: R=0.45, R^2^=0.20; healthy functional connectome: R=0.34, R^2^=0.12 |
| Modulation of Nigrofugal and Pallidofugal Pathways in Deep Brain Stimulation for Parkinson Disease.[(17)](https://paperpile.com/c/JBEekg/CwmSI) | PD | Motor improvement, medication dose | Structural (disease-specific PPMI: n=43) | N/A |
| Connectivity profile of thalamic deep brain stimulation to effectively treat essential tremor.[(18)](https://paperpile.com/c/JBEekg/hdPse) | ET | Motor improvement, side-effects | Structural (healthy subjects: n=20);  Functional (healthy subjects: n=1000) | healthy structural connectome: R=0.40, R^2^=0.16; healthy functional connectome: R=0.36, R^2^=0.13 |
| Unilateral Thalamic Deep Brain Stimulation for Voice Tremor.[(19)](https://paperpile.com/c/JBEekg/o75wb) | ET | Motor improvement (voice tremor score) | Structural (healthy subjects: n=72) | N/A |
| Functional and Structural Connectivity Patterns Associated with Clinical Outcomes in Deep Brain Stimulation of the Globus Pallidus Internus for Generalized Dystonia.[(20)](https://paperpile.com/c/JBEekg/fBYTM) | Dystonia | Motor improvement | Structural (healthy subjects: n=32); Functional (healthy subjects: n=1000) | N/A |
| Left Prefrontal Connectivity Links Subthalamic Stimulation with Depressive Symptoms.[(21)](https://paperpile.com/c/JBEekg/ulu0E) | PD | Mood change | Structural (disease-specific PPMI: n=90) | R=0.26, R^2^=0.07 |
| Connectivity Patterns of Subthalamic Stimulation Influence Pain Outcomes in Parkinson’s Disease.[(22)](https://paperpile.com/c/JBEekg/uiJzC) | PD | Pain improvement | Structural | R=0.68, R^2^=0.46 |
| The structural connectivity of subthalamic deep brain stimulation correlates with impulsivity in Parkinson’s disease.[(23)](https://paperpile.com/c/JBEekg/M4qKw) | PD | Neuropsychiatric side-effects (impulsivity, compulsivity, etc.) | Structural (disease-specific PPMI: n=90) | N/A |
| Functional segregation of basal ganglia pathways in Parkinson’s disease.[(24)](https://paperpile.com/c/JBEekg/EHwGx) | PD | Reaction time | Structural (disease-specific PPMI: n=90) | R=0.59, R^2^=0.35 |
| Management of Pisa syndrome with lateralized subthalamic stimulation.[(25)](https://paperpile.com/c/JBEekg/11X8J) | PD | Pisa syndrome | Structural (healthy subjects: n=985) | N/A |
| Subthalamic neuromodulation improves short-term motor learning in Parkinson’s disease.[(26)](https://paperpile.com/c/JBEekg/5Qk3Q) | PD | Motor learning | Functional (healthy subjects: n=1000) | R=0.43, R^2^=0.18 |
| Towards a comprehensive pipeline for deep brain stimulation imaging.[(16)](https://paperpile.com/c/JBEekg/TzsxI) | PD | Motor improvement | Structural (disease-specific PPMI: n=90) | R=0.53, R^2^=0.28 |
| Localizing parkinsonism based on focal brain lesions.[(27)](https://paperpile.com/c/JBEekg/bfUcd) | PD | Motor improvement | Functional (healthy subjects: n=1000) | R=0.29, R^2^=0.08 |
| Probabilistic Mapping of Deep Brain Stimulation: Insights from 15 Years of Therapy.[(28)](https://paperpile.com/c/JBEekg/jcFHd) | PD, dystonia, ET | Motor improvement | Structural (healthy subjects: n=985) & Functional (healthy subjects: n=1000) | N/A |
| Mapping efficacious deep brain stimulation for pediatric dystonia.[(29)](https://paperpile.com/c/JBEekg/SdaoP) | Dystonia (pediatric) | Motor improvement | Structural (healthy subjects: n=985) &  Functional (healthy subjects: n=1000) | N/A |
| Case Report: Globus Pallidus Internus (GPi) Deep Brain Stimulation Induced Keyboard Typing Dysfunction [(30)](https://paperpile.com/c/JBEekg/drV97) | Dystonia | Keyboard typing dysfunction | Structural (healthy subjects; n=842) | N/A |
| Deep Brain Stimulation of the Internal Pallidum in Lesch--Nyhan Syndrome: Clinical Outcomes and Connectivity Analysis.[(31)](https://paperpile.com/c/JBEekg/KYoiI) | Lesch-Nyhan syndrome | Motor improvement , self-harm behaviour improvement | Structural (healthy subjects: n=32) | R=0.96, R^2^=0.92 |
| PSA and VIM DBS efficiency in essential tremor depends on distance to the dentatorubrothalamic tract.[(32)](https://paperpile.com/c/JBEekg/hceNn) | ET | Motor outcome | Structural (healthy subjects: n=32) | R=0.66, R^2^=0.44 |
| Normative vs. patient-specific brain connectivity in deep brain stimulation.[(33)](https://paperpile.com/c/JBEekg/330R1) | PD | Motor improvement | Structural × 2 (disease-specific PPMI: n=85; healthy subjects: n=32) | disease-specific connectome: R=0.25, R^2^=0.06; healthy connectome: R=0.31, R^2^=0.10 |
| Parkinson’s disease motor subtypes and bilateral GPi deep brain stimulation: One-year outcomes.[(34)](https://paperpile.com/c/JBEekg/hkH3r) | PD | Dyskinesia side-effects | Structural (n=32 healthy subjects subjects);  Functional (n=1000 healthy subjects subjects) | N/A |
| Deep brain stimulation: Imaging on a group level.[(35)](https://paperpile.com/c/JBEekg/SKVrY) | PD | Motor improvement | Structural (PPMI: n=85) | R=-0.4-0.46, R^2^=0.07 |
| Network Fingerprint of Stimulation-Induced Speech Impairment in Essential Tremor.[(36)](https://paperpile.com/c/JBEekg/zC0L9) | ET | Dysarthria side-effects, motor improvement | Structural (healthy subjects: n=20) | R=0.79, R^22^=0.62 |

**n* denotes the number of individual subject data used to create the connectome. Abbreviations: *DBS =* deep brain stimulation; *ET* = essential tremor; PD = Parkinson’s disease; *PPMI* = Parkinson’s Progression Markers Initiative

**Supplementary Table 2. MRI acquisition parameters.**

| **Sequence** | **MRI manufacturer** | **Field strength (T)** | **Matrix** | **FOV (mm)** | **Slice thickness (mm)** | **TE (msec)** | **TR (msec)** | **T1 (msec)** | **Flip angle (degree)** | **Number of volumes** |
| --- | --- | --- | --- | --- | --- | --- | --- | --- | --- | --- |
| T1 | Signa Excite, GE | 1.5 | 256 x 256 | 358 | 1.4 | 5.3 | 12.4 | 300 | 20 | N/A |
| T1 | Signa HDxt | 3 | 320 x 320 | 320 | 1 | 3.7 | 9 | 450 | 12 | N/A |
| rsfMRI | Signa Excite, GE | 1.5 | 64 x 64 | 288 | 4.5 | 35 | 2000 | 0 | 85 | 180 |
| rsfMRI | Signa HDxt | 3 | 64 x 64 | 256 | 4 | 30 | 2200 | 0 | 85 | 180 |

Abbreviations: FOV = field of view; kHz = kilohertz; T = tesla; rsfMRI = resting state functional magnetic resonance imaging; TE = echo time; TR = repetition time; T1 = T1-weighted; mm = millimetre; msec = milliseconds; MRI = magnetic resonance imaging.

**Supplementary Table 3. Demographic information of PD patients used for VTA analysis.**

|  | **Age (years)** | **Sex** | **Disease duration (years)** | **Pre-op MDS-UPDRS** | **Pre-op Levodopa equivalent (mg/day)** | **Active contact coordinates (mm)** |
| --- | --- | --- | --- | --- | --- | --- |
| VTA cohort (n=50) | 59 ± 7 | 30M  20F | 11 ± 4 | 43 ± 12 | 1616 ± 725 | 12 ± 2, -13 ± 2, -5 ± 3 |

Values represent the mean and standard deviation. To calculate mean active contact coordinates, left sided contacts were flipped to the right. Abbreviations: DBS = deep brain stimulation; F = female; M = male; MDS-UPDRS = Movement Disorders Society - Unified Parkinson’s Disease Rating Scale; mg = milligram; PD = Parkinson’s disease; VTA = volume of tissue activated


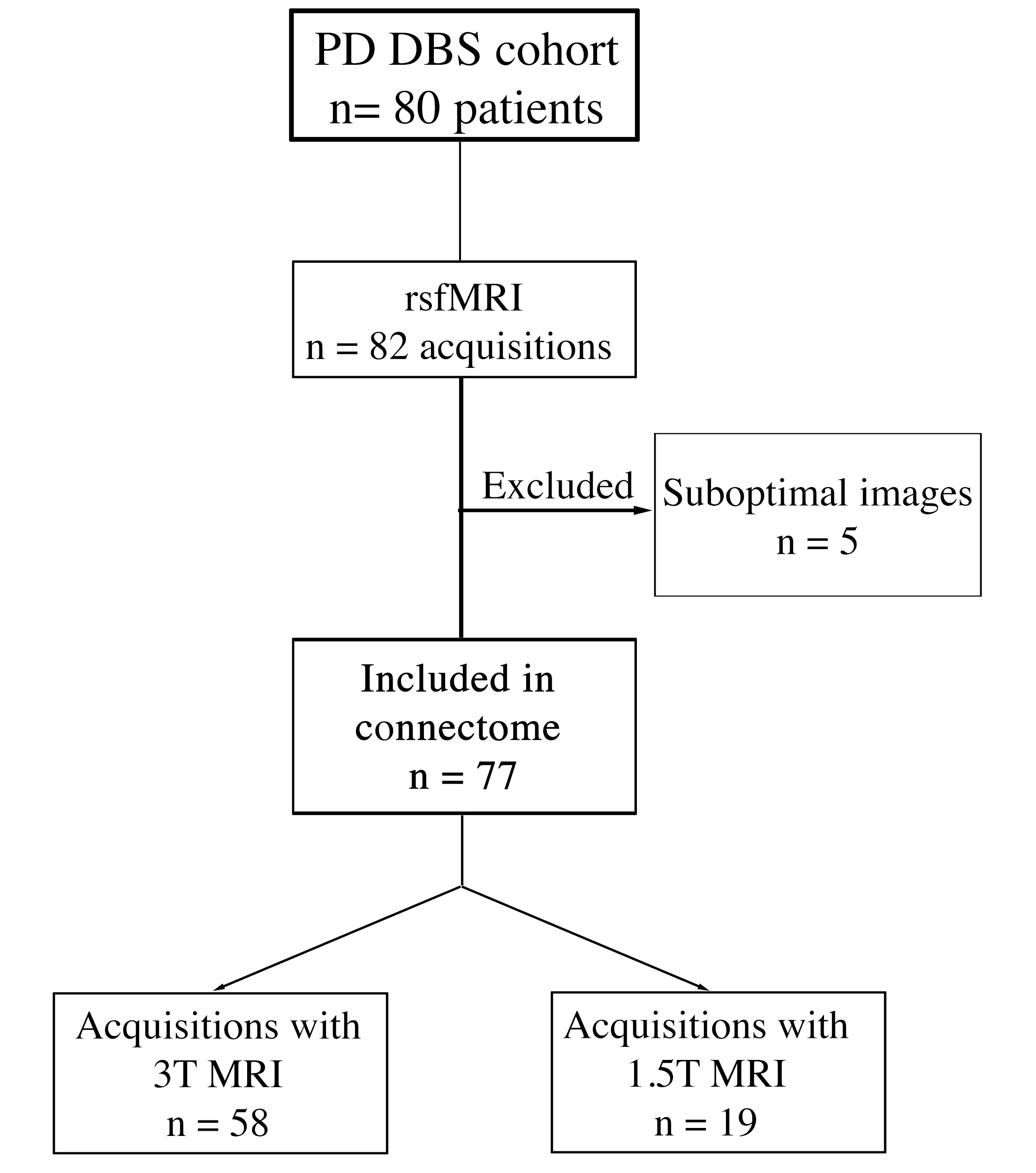


**Supplementary Fig. 1. Flowchart of patients included in the Tor-PD connectome.** Two of the 80 patients scanned underwent rsfMRI twice, resulting in a total of 82 rsfMRI acquisitions. Abbreviations: DBS = deep brain stimulation; MRI = magnetic resonance imaging; n = number; PD = Parkinson’s disease; rsfMRI = resting-state functional magnetic resonance imaging; T = tesla.


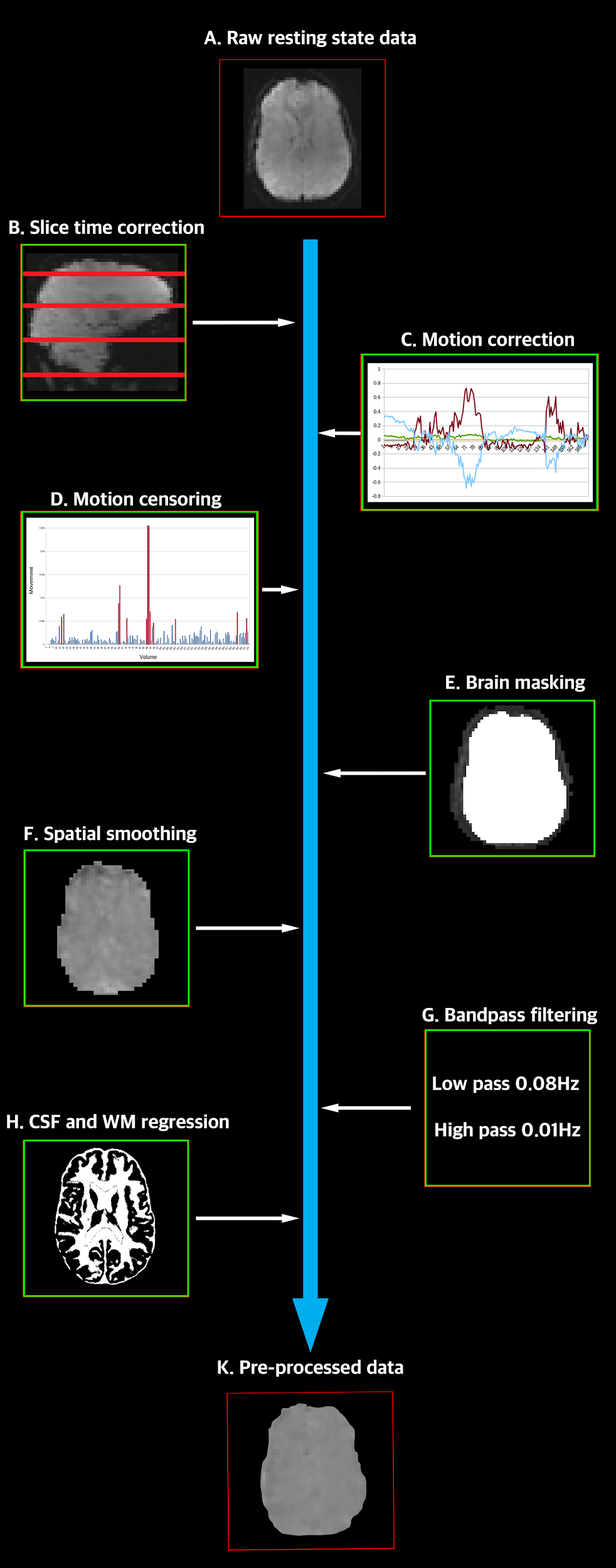


**Supplementary Fig. 2. fMRI preprocessing pipeline.** A schematic showing the preprocessing steps that were undertaken for each resting-state fMRI acquisition prior to inclusion in the Tor-PD connectome. Panel A: Raw rsfMRI data. Panel B: Slice time correction applied in interleaved fashion. Panel C: Motion correction of the rsfMRI time series. Panel D: Motion censoring of rsfMRI volumes corrupted by excessive motion. Panel E: Brain masking to exclude cranial and extracranial data. Panel F: Spatial smoothing with gaussian kernel of 6mm FWHM. Panel G: Bandpass filtering with high pass filter of 0.01Hz and a low pass filter of 0.08Hz. Panel H: Regression of the average BOLD time series over CSF and WM. Abbreviations: BOLD = blood-oxygen-level-dependent; CSF = cerebrospinal fluid; fMRI = functional magnetic resonance imaging; FWHM = full width half maximum; Hz = hertz; mm = millimeter; rsfMRI = resting state functional magnetic resonance imaging; WM = white matter.


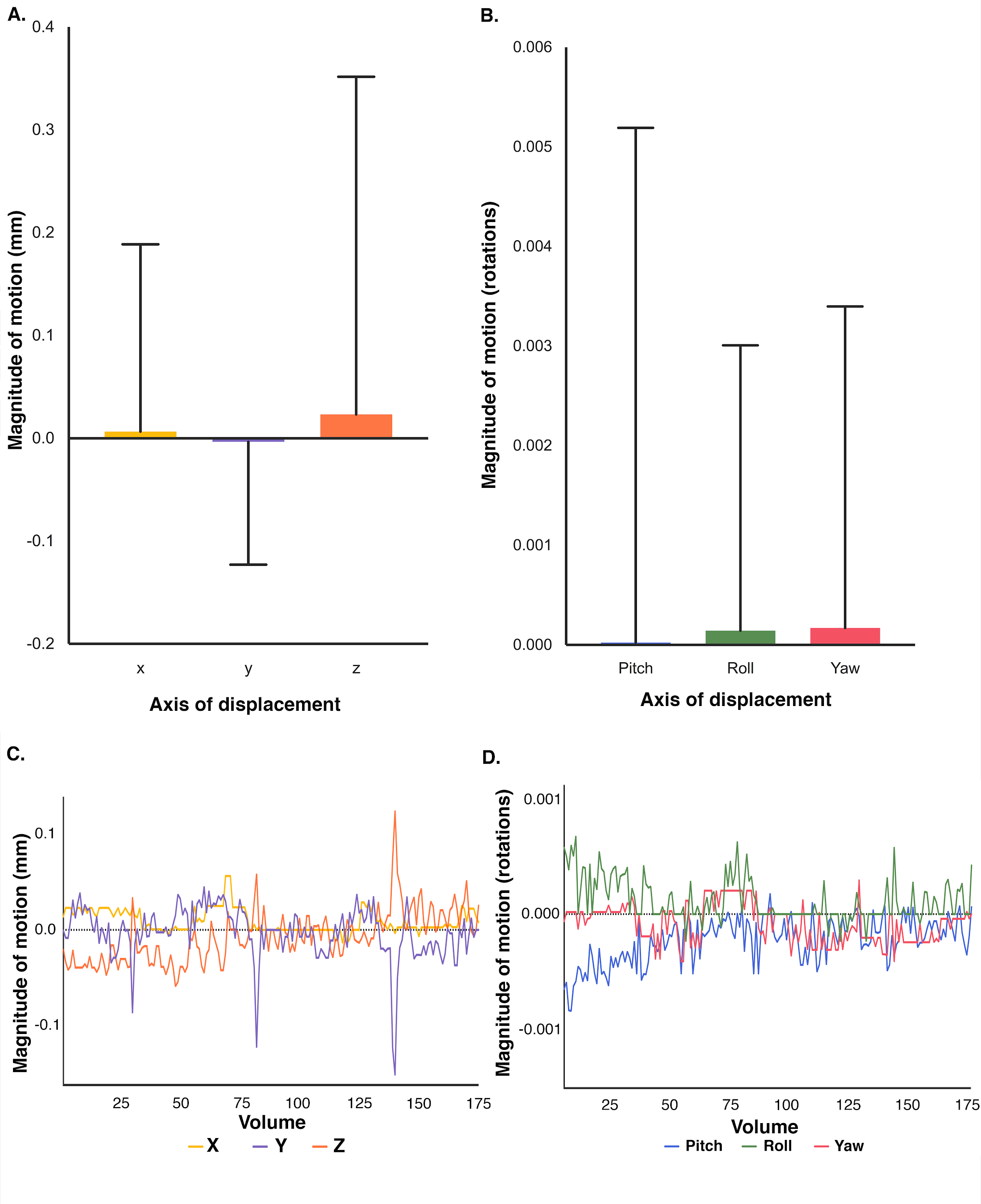


**Supplemental Fig. 3. MRI motion.** The mean motion of all patients included in the Tor-PD connectome during rsfMRI acquisitions. The magnitude of motion in x, y, and z axes are shown in A, while the magnitude of motion in pitch, roll, and yaw axes are shown in B. Error bars denote the standard deviation. Graphs C. and D. show the motion of an individual patient included in the Tor-PD connectome, across all volumes of a single rsfMRI acquisition. The magnitude of motion in x, y, and z axes are shown in C, while the magnitude of motion in pitch, roll, and yaw axes are shown in D. Abbreviations: mm = millimetre; rsfMRI = resting state functional magnetic resonance imaging.

**Supplementary Fig. 4. NaN mask.** A mask corresponding to the voxels in MNI152 standard that were less than 20% NaN across the 77 BOLD signal time-series matrices comprising the Tor-PD connectome. Abbreviations: BOLD = blood-oxygen-level-dependent; MNI = Montreal Neurological Institute.
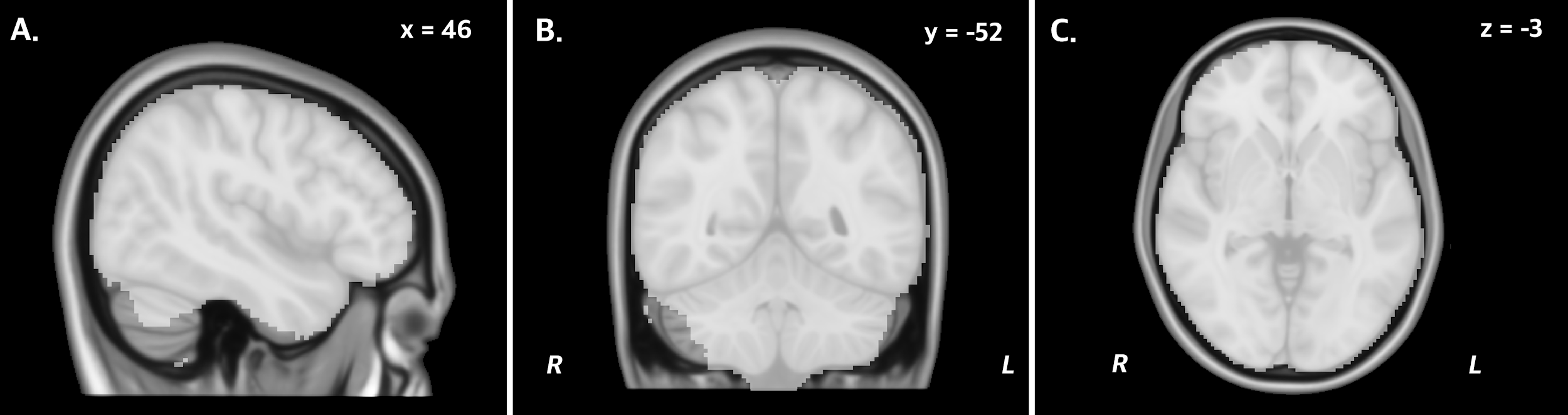


References

1. [Yeo BTT, Krienen FM, Sepulcre J, Sabuncu MR, Lashkari D, Hollinshead M, Roffman JL, Smoller JW, Zöllei L, Polimeni JR, et al. The organization of the human cerebral cortex estimated by intrinsic functional connectivity. *J Neurophysiol* (2011) **106**:1125–1165.](http://paperpile.com/b/JBEekg/mnsqN)

2. [Jenkinson M, Beckmann CF, Behrens TEJ, Woolrich MW, Smith SM. FSL. *Neuroimage* (2012) **62**:782–790.](http://paperpile.com/b/JBEekg/WWlSf)

3. [Smith SM, Jenkinson M, Woolrich MW, Beckmann CF, Behrens TEJ, Johansen-Berg H, Bannister PR, De Luca M, Drobnjak I, Flitney DE, et al. Advances in functional and structural MR image analysis and implementation as FSL. *Neuroimage* (2004) **23 Suppl 1**:S208–19.](http://paperpile.com/b/JBEekg/T8nnO)

4. [Cox RW. AFNI: software for analysis and visualization of functional magnetic resonance neuroimages. *Comput Biomed Res* (1996) **29**:162–173.](http://paperpile.com/b/JBEekg/Y2icG)

5. [Jenkinson M, Bannister P, Brady M, Smith S. Improved Optimization for the Robust and Accurate Linear Registration and Motion Correction of Brain Images. *Neuroimage* (2002) **17**:825–841.](http://paperpile.com/b/JBEekg/wdPVm)

6. [Power JD, Barnes KA, Snyder AZ, Schlaggar BL, Petersen SE. Spurious but systematic correlations in functional connectivity MRI networks arise from subject motion. *Neuroimage* (2012) **59**:2142–2154.](http://paperpile.com/b/JBEekg/krVlo)

7. [Caballero-Gaudes C, Reynolds RC. Methods for cleaning the BOLD fMRI signal. *Neuroimage* (2017) **154**:128–149.](http://paperpile.com/b/JBEekg/tSo3W)

8. [Fischl B, Salat DH, Busa E, Albert M, Dieterich M, Haselgrove C, van der Kouwe A, Killiany R, Kennedy D, Klaveness S, et al. Whole brain segmentation: automated labeling of neuroanatomical structures in the human brain. *Neuron* (2002) **33**:341–355.](http://paperpile.com/b/JBEekg/NnEnb)

9. [Fischl B, van der Kouwe A, Destrieux C, Halgren E, Ségonne F, Salat DH, Busa E, Seidman LJ, Goldstein J, Kennedy D, et al. Automatically parcellating the human cerebral cortex. *Cereb Cortex* (2004) **14**:11–22.](http://paperpile.com/b/JBEekg/FvjpR)

10. [Horn A, Reich M, Vorwerk J, Li N, Wenzel G, Fang Q, Schmitz-Hübsch T, Nickl R, Kupsch A, Volkmann J, et al. Connectivity Predicts deep brain stimulation outcome in Parkinson disease. *Ann Neurol* (2017) **82**:67–78.](http://paperpile.com/b/JBEekg/s1LM6)

11. [Elias GJB, De Vloo P, Germann J, Boutet A, Gramer RM, Joel SE, Morlion B, Nuttin B, Lozano AM. Mapping the network underpinnings of central poststroke pain and analgesic neuromodulation. *Pain* (2020) doi:](http://paperpile.com/b/JBEekg/LgtKd)[10.1097/j.pain.0000000000001998](http://dx.doi.org/10.1097/j.pain.0000000000001998)

12. [Li N, Baldermann JC, Kibleur A, Treu S, Akram H, Elias GJB, Boutet A, Lozano AM, Al-Fatly B, Strange B, et al. A unified connectomic target for deep brain stimulation in obsessive-compulsive disorder. *Nat Commun* (2020) **11**:3364.](http://paperpile.com/b/JBEekg/5fOne)

13. [Boutet A, Jain M, Elias GJB, Gramer R, Germann J, Davidson B, Coblentz A, Giacobbe P, Kucharczyk W, Wennberg RA, et al. Network Basis of Seizures Induced by Deep Brain Stimulation: Literature Review and Connectivity Analysis. *World Neurosurg* (2019) **132**:314–320.](http://paperpile.com/b/JBEekg/IAV24)

14. [Yan H, Boutet A, Mithani K, Germann J, Elias GJB, Yau I, Go C, Kalia SK, Lozano AM, Fasano A, et al. Aggressiveness after centromedian nucleus stimulation engages prefrontal thalamocortical circuitry. *Brain Stimul* (2020) **13**:357–359.](http://paperpile.com/b/JBEekg/XQdtC)

15. [Elias GJB, Giacobbe P, Boutet A, Germann J, Beyn ME, Gramer RM, Pancholi A, Joel SE, Lozano AM. Probing the circuitry of panic with deep brain stimulation: Connectomic analysis and review of the literature. *Brain Stimul* (2020) **13**:10–14.](http://paperpile.com/b/JBEekg/LOs52)

16. [Horn A, Li N, Dembek TA, Kappel A, Boulay C, Ewert S, Tietze A, Husch A, Perera T, Neumann W-J, et al. Lead-DBS v2: Towards a comprehensive pipeline for deep brain stimulation imaging. *Neuroimage* (2019) **184**:293–316.](http://paperpile.com/b/JBEekg/TzsxI)

17. [Avecillas-Chasin JM, Honey CR. In Reply: Modulation of Nigrofugal and Pallidofugal Pathways in Deep Brain Stimulation for Parkinson Disease. *Neurosurgery* (2020) **87**:E423–E424.](http://paperpile.com/b/JBEekg/CwmSI)

18. [Al-Fatly B, Ewert S, Kübler D, Kroneberg D, Horn A, Kühn AA. Connectivity profile of thalamic deep brain stimulation to effectively treat essential tremor. *Brain* (2019) **142**:3086–3098.](http://paperpile.com/b/JBEekg/hdPse)

19. [Avecillas-Chasin JM, Poologaindran A, Morrison MD, Rammage LA, Honey CR. Unilateral Thalamic Deep Brain Stimulation for Voice Tremor. *Stereotact Funct Neurosurg* (2018) **96**:392–399.](http://paperpile.com/b/JBEekg/o75wb)

20. [Okromelidze L, Tsuboi T, Eisinger RS, Burns MR, Charbel M, Rana M, Grewal SS, Lu C-Q, Almeida L, Foote KD, et al. Functional and Structural Connectivity Patterns Associated with Clinical Outcomes in Deep Brain Stimulation of the Globus Pallidus Internus for Generalized Dystonia. *AJNR Am J Neuroradiol* (2020) **41**:508–514.](http://paperpile.com/b/JBEekg/fBYTM)

21. [Irmen F, Horn A, Mosley P, Perry A, Petry-Schmelzer JN, Dafsari HS, Barbe M, Visser-Vandewalle V, Schneider G-H, Li N, et al. Left Prefrontal Connectivity Links Subthalamic Stimulation with Depressive Symptoms. *Ann Neurol* (2020) **87**:962–975.](http://paperpile.com/b/JBEekg/ulu0E)

22. [Cury RG, Teixeira MJ, Galhardoni R, Silva V, Iglesio R, França C, Arnaut D, Fonoff ET, Barbosa ER, Ciampi de Andrade D. Connectivity Patterns of Subthalamic Stimulation Influence Pain Outcomes in Parkinson’s Disease. *Front Neurol* (2020) **11**:9.](http://paperpile.com/b/JBEekg/uiJzC)

23. [Mosley PE, Paliwal S, Robinson K, Coyne T, Silburn P, Tittgemeyer M, Stephan KE, Perry A, Breakspear M. The structural connectivity of subthalamic deep brain stimulation correlates with impulsivity in Parkinson’s disease. *Brain* (2020) **143**:2235–2254.](http://paperpile.com/b/JBEekg/M4qKw)

24. [Neumann W-J, Schroll H, de Almeida Marcelino AL, Horn A, Ewert S, Irmen F, Krause P, Schneider G-H, Hamker F, Kühn AA. Functional segregation of basal ganglia pathways in Parkinson’s disease. *Brain* (2018) **141**:2655–2669.](http://paperpile.com/b/JBEekg/EHwGx)

25. [Lizarraga KJ, Naghibzadeh M, Boutet A, Elias GJB, Fasano A. Management of Pisa syndrome with lateralized subthalamic stimulation. *J Neurol* (2018) **265**:2442–2444.](http://paperpile.com/b/JBEekg/11X8J)

26. [de Almeida Marcelino AL, Horn A, Krause P, Kühn AA, Neumann W-J. Subthalamic neuromodulation improves short-term motor learning in Parkinson’s disease. *Brain* (2019) **142**:2198–2206.](http://paperpile.com/b/JBEekg/5Qk3Q)

27. [Joutsa J, Horn A, Hsu J, Fox MD. Localizing parkinsonism based on focal brain lesions. *Brain* (2018) **141**:2445–2456.](http://paperpile.com/b/JBEekg/bfUcd)

28. [Elias GJB, Boutet A, Joel SE, Germann J, Gwun D, Neudorfer C, Gramer RM, Algarni M, Paramanandam V, Prasad S, et al. Probabilistic Mapping of Deep Brain Stimulation: Insights from 15 Years of Therapy. *Ann Neurol* (2020) doi:](http://paperpile.com/b/JBEekg/jcFHd)[10.1002/ana.25975](http://dx.doi.org/10.1002/ana.25975)

29. [Coblentz A, Elias GJB, Boutet A, Germann J, Algarni M, Oliveira LM, Neudorfer C, Widjaja E, Ibrahim GM, Kalia SK, et al. Mapping efficacious deep brain stimulation for pediatric dystonia. *J Neurosurg Pediatr* (2021)1–11.](http://paperpile.com/b/JBEekg/SdaoP)

30. [Wong JK, Armstrong MJ, Almeida L, Wagle Shukla A, Patterson A, Okun MS, Malaty IA. Case Report: Globus Pallidus Internus (GPi) Deep Brain Stimulation Induced Keyboard Typing Dysfunction. *Front Hum Neurosci* (2020) **14**:583441.](http://paperpile.com/b/JBEekg/drV97)

31. [Tambirajoo K, Furlanetti L, Hasegawa H, Raslan A, Gimeno H, Lin J-P, Selway R, Ashkan K. Deep Brain Stimulation of the Internal Pallidum in Lesch--Nyhan Syndrome: Clinical Outcomes and Connectivity Analysis. *Neuromodulation: Technology at the Neural Interface* (2020) Available at:](http://paperpile.com/b/JBEekg/KYoiI) <https://onlinelibrary.wiley.com/doi/abs/10.1111/ner.13217>

32. [Dembek TA, Petry-Schmelzer JN, Reker P, Wirths J, Hamacher S, Steffen J, Dafsari HS, Hövels M, Fink GR, Visser-Vandewalle V, et al. PSA and VIM DBS efficiency in essential tremor depends on distance to the dentatorubrothalamic tract. *Neuroimage Clin* (2020) **26**:102235.](http://paperpile.com/b/JBEekg/hceNn)

33. [Wang Q, Akram H, Muthuraman M, Gonzalez-Escamilla G, Sheth SA, Oxenford S, Yeh F-C, Groppa S, Vanegas-Arroyave N, Zrinzo L, et al. Normative vs. patient-specific brain connectivity in deep brain stimulation. *Neuroimage* (2021) **224**:117307.](http://paperpile.com/b/JBEekg/330R1)

34. [Tsuboi T, Lemos Melo Lobo Jofili Lopes J, Patel B, Legacy J, Moore K, Eisinger RS, Almeida L, Foote KD, Okun MS, Ramirez-Zamora A. Parkinson’s disease motor subtypes and bilateral GPi deep brain stimulation: One-year outcomes. *Parkinsonism Relat Disord* (2020) **75**:7–13.](http://paperpile.com/b/JBEekg/hkH3r)

35. [Treu S, Strange B, Oxenford S, Neumann W-J, Kühn A, Li N, Horn A. Deep brain stimulation: Imaging on a group level. *Neuroimage* (2020) **219**:117018.](http://paperpile.com/b/JBEekg/SKVrY)

36. [Petry-Schmelzer JN, Jergas H, Thies T, Steffen JK, Reker P, Dafsari HS, Mücke D, Fink GR, Visser-Vandewalle V, Dembek TA, et al. Network Fingerprint of Stimulation-Induced Speech Impairment in Essential Tremor. *Ann Neurol* (2020) doi:](http://paperpile.com/b/JBEekg/zC0L9)[10.1002/ana.25958](http://dx.doi.org/10.1002/ana.25958)
